# Human Tyrosyl-DNA Phosphodiesterase 1 Possesses Transphosphooligonucleotidation Activity With Primary Alcohols

**DOI:** 10.3389/fcell.2020.604732

**Published:** 2020-12-23

**Authors:** Nadezhda Dyrkheeva, Rashid Anarbaev, Natalia Lebedeva, Maxim Kuprushkin, Alexandra Kuznetsova, Nikita Kuznetsov, Nadejda Rechkunova, Olga Lavrik

**Affiliations:** ^1^Institute of Chemical Biology and Fundamental Medicine, Siberian Branch of the Russian Academy of Sciences, Novosibirsk, Russia; ^2^Department of Natural Sciences, Novosibirsk State University, Novosibirsk, Russia

**Keywords:** tyrosyl-DNA phosphodiesterase 1, 3′-phosphoglycolate, glycerol, alcohol, ethanol, spinocerebellar ataxia with axonal neuropathy type 1, DNA damage, DNA repair

## Abstract

Human tyrosyl-DNA phosphodiesterase 1 (TDP1) belongs to the phospholipase D superfamily, whose members contain paired catalytic histidine and lysine residues within two conserved motifs and hydrolyze phosphodiester bonds. TDP1 is a DNA repair enzyme that processes 3′ DNA end blocking lesions and a wide range of synthetic DNA adducts as a substrate. TDP1 hydrolyzes DNA-adducts via two coordinated S_N_2 nucleophilic attacks mediated by the action of two histidine residues and leads to the formation of the covalent intermediate. Hydrolysis of this intermediate is proposed to be carried out by a water molecule that is activated by the His493 residue acting as a general base. It was known that phospholipase D enzymes are able to catalyze not only hydrolysis but also a transphosphatidylation reaction in the presence of primary alcohols in which they transfer the substrate to the alcohol instead of water. Here, we first demonstrated that TDP1 is able to undergo a “transphosphooligonucleotidation” reaction, transferring the substrate residue to the alcohol, thus inducing the formation of covalent DNA adducts with different primary alcohol residues. Such adducts can be accumulated in the conditions of high concentration of alcohol. We demonstrated that glycerol residue was efficiently cleaved from the 3′-end by TDP1 but not by its mutant form associated with the disease spinocerebellar ataxia with axonal neuropathy. Therefore, the second reaction step can be carried out not only by a water molecule but also by the other small nucleophilic molecules, e.g., glycerol and ethanol. Thus, in some cases, TDP1 can be regarded not only as a repair enzyme but also as a source of DNA damage especially in the case of mutation. Such damages can make a negative contribution to the stability of cell vitality.

## Introduction

Tyrosyl-DNA phosphodiesterase 1 (TDP1) is an enzyme of the phospholipase D (PLD) superfamily (Interthal et al., 2001). Phospholipases hydrolyze phospholipids into fatty acids and other lipophilic substances. There are four major classes of phospholipases (A, B, C, and D) segregated by the catalyzed reaction type. PLD superfamily enzymes possess phosphodiesterase activity. Hydrolysis of the abundant membrane phospholipid phosphatidylcholine with generation of choline and phosphatidic acid is the most commonly studied reaction of PLD enzymes. It is known that the PLD superfamily plays a central role in a variety of functions in prokaryotes, viruses, yeasts, fungi, plants, and eukaryotic species. Phosphatidic acid generated by PLD participates in vesicular trafficking, exocytosis, autophagy, and regulation of cellular metabolism, cytoskeletal reorganization, and tumorigenesis. PLD is a regulator of membrane remodeling, intercellular signaling, protein trafficking, and metabolic pathways; it may play a role in multiple sclerosis, cardiovascular, neurodegenerative, and infectious diseases, and in cell motility and migration, a critical step in the spread of cancer (Peng and Frohman, 2012; Bruntz et al., 2014; Frohman, 2015).

Historically, bacterial virulence factors that demonstrated the release of a choline were named PLDs for the function. Then, it was found that PLD enzymes can hydrolyze not only phosphatidylcholine but also other glycerophospholipids. In addition to hydrolyzing phospholipids, PLDs are able to catalyze a transphosphatidylation reaction in the presence of primary alcohols in which the phosphatidyl group from the hydrolysis of phosphatidylcholine is transferred to the alcohol instead of water, thus conducting headgroup exchange on phosphatidic acid at the terminal phosphodiester bond (Selvy et al., 2011).

PLD family members include not only phospholipases but also nucleases (Koonin, 1996; Ponting and Kerr, 1996) that contain HKD motifs (HxKxxxxD, where x is any amino acid) and hydrolyze phosphodiester bonds via a similar reaction mechanism (Koonin, 1996; Ponting and Kerr, 1996; Interthal et al., 2001; Selvy et al., 2011; Bruntz et al., 2014). TDP1 was the first eukaryotic PLD for which the crystal structure was obtained in 2002 by Davies et al. (2002b). TDP1 enzymatic activity was first found in yeast *Saccharomyces cerevisiae* as repairing the covalently linked adducts of DNA topoisomerase I (TOP1) by catalyzing the hydrolysis of the phosphodiester bond between the tyrosine residue of TOP1 peptide and the 3′ phosphate of DNA. The result DNA product has a break with 3′ phosphate and 5′ hydroxyl groups (Yang et al., 1996; Pouliot et al., 1999). TDP1 possesses a unique HKD motif that differs from other PLD superfamily members, and its orthologs represent a distinct class within the PLD superfamily. TDP1 catalytic center contains two histidine residues His493 and His263 (Interthal et al., 2001). The His493Arg mutation in Tdp1 gene causes spinocerebellar ataxia with axonal neuropathy type 1 (SCAN1) by affecting neuronal cells (Takashima et al., 2002). TDP1 activity is not limited by the removal of cellular TOP1 adducts. TDP1 was shown to catalyze 3′ phosphoglycolate removal from a single-stranded oligonucleotide and a single strand overhangs of DNA double-strand breaks (Inamdar et al., 2002; Raymond et al., 2005). TDP1 is now regarded as a general 3′ DNA end-processing enzyme that acts within the single-strand break repair complex to remove adducts and to prepare the DNA ends bearing 3′ phosphate group for further processing by DNA repair enzymes (Rass et al., 2007). TDP1 also possesses a DNA and RNA 3′-nucleosidase activity that removes from the 3′-end of the substrate a single nucleoside, as well as nucleoside analogs terminating DNA synthesis and widely used as antiviral and anticancer agents and a variety of synthetic DNA adducts for example with molecules, such as biotin and various fluorophores (Dexheimer et al., 2008; Murai et al., 2012; Huang et al., 2013; Dyrkheeva et al., 2018; Brettrager and van Waardenburg, 2019). TDP1 can also process other 3′ DNA end blocking lesions as a substrate: 3′ abasic sites (tetrahydrofuran and α,β-unsaturated aldehyde) and different bulky substituents (Hawkins et al., 2009; Interthal et al., 2005a; Zhou et al., 2005). TDP1 can reverse not only 3′-TOP1-DNA cross-linked bonds but also it is able to release different DNA-protein cross-links. It was found that both human and yeast TDP1 proteins have the ability to process 5′-phosphotyrosyl and 5′-phosphotyrosyl-linked peptide substrates, thus indicating that they can hydrolyze covalently linked adducts of DNA with TOP2 (Nitiss et al., 2006; Murai et al., 2012; Zhang et al., 2020). It also works on the other large adducts including protein fragments (peptides) as a result of failed Schiff base linked proteins, such as proteolytically processed poly(ADP-ribose) polymerase 1 (PARP1)-DNA adducts. These different protein-DNA adducts can be stabilized by chemotherapeutic compounds, e.g., camptothecins, etoposide, and local DNA perturbations introduced by irradiation and endogenous reactive oxygen species (Brettrager and van Waardenburg, 2019).

We have previously shown that human TDP1 can also cleave an apurinic/apyrimidinic (AP) site and its synthetic analogs located inside DNA strand with the formation of 3′ phosphate termini. This observation allows suggesting a novel pathway of AP site repair independent of AP endonuclease 1 (APE1) (Lebedeva et al., 2011, 2012, 2013; Kuznetsov et al., 2017). In contrast to APE1, TDP1 more effectively hydrolyzes AP sites in single-stranded DNA than in DNA duplex (Lebedeva et al., 2012). This suggests that TDP1 may be involved in the repair of AP sites in single-stranded genomic DNA regions that occur in all the major processes of DNA metabolism: replication, transcription, recombination, and repair.

We revealed that no cleavage product was detected for natural AP site in the case of SCAN1 (Lebedeva et al., 2012), whereas non-nucleotide insertions mimicking the AP site were cleaved by this mutant, although with lower efficiency than by wild-type (WT) TDP1 (Lebedeva et al., 2015; Kuznetsov et al., 2017). Moreover, we found that in the reaction catalyzed by SCAN1, two bands were observed on the gel when DNA with synthetic AP site mimetics was used as the substrate. One band corresponded to TDP1 cleavage product, whereas the second one migrated slower in the gel. As TDP1 belongs to the PLD family, we suggested that it can catalyze an equivalent “transferring” reaction in the presence of primary alcohols. Here, we analyze the second reaction product and reveal evidently that it corresponds to DNA fragment with 3′ phosphoglycerol residue. It can be generated because glycerol acts as a nucleophile on the second step of the reaction. Thus, we first demonstrated that TDP1, the same as other PLD enzymes, is able to undergo a reaction transferring the substrate residue to the alcohol instead of water. Also, we could see this second product on the gel with other alcohols including ethanol. This product is generated by SCAN1 and WT TDP1 in the presence of high alcohol concentration. Therefore, the second reaction step can be carried out not only by a water molecule but also by the other small nucleophilic molecules, e.g., glycerol and ethanol.

## Results

### Tyrosyl-DNA Phosphodiesterase 1 Wild Type and His493Arg Tyrosyl-DNA Phosphodiesterase 1 Mutant (SCAN1) Activity on Hairpin Substrate Containing Non-nucleotide Insertion

**FIGURE 1:**
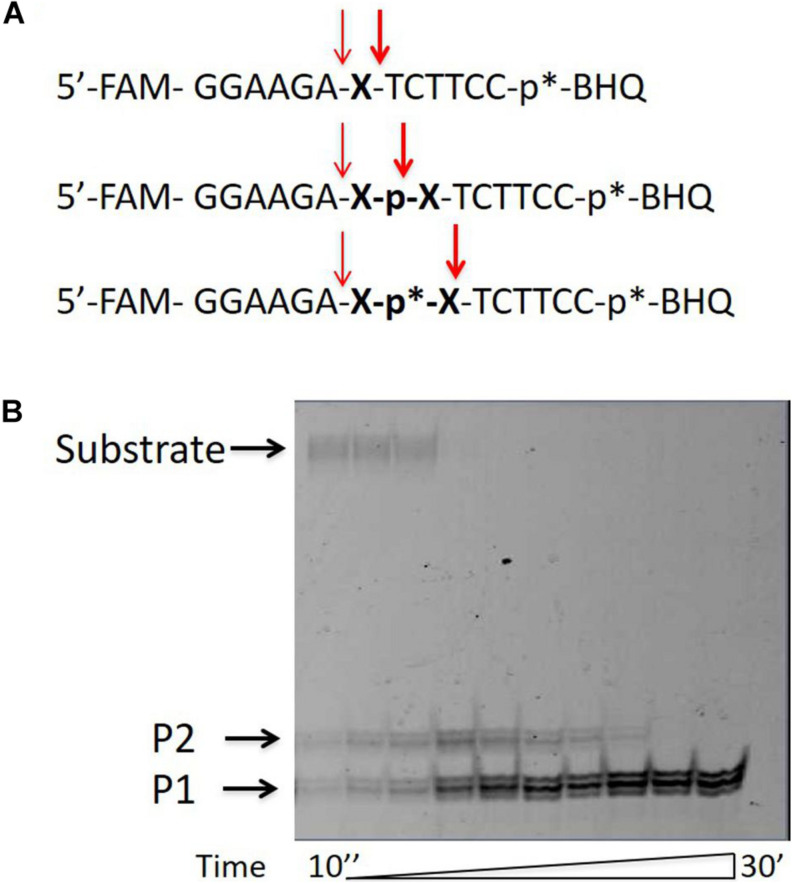
Tyrosyl-DNA phosphodiesterase 1 (TDP1) mutant [spinocerebellar ataxia with axonal neuropathy type 1 (SCAN1)] activity on hairpin substrate containing non-nucleotide insertion. **(A)** Oligonucleotide substrates. BHQ1, Black Hole Quencher 1; FAM, 5(6)-carboxyfluorescein label; non-nucleotide insertions (X); p*, 1,3-dimethyl-2-(phosphorylimino)imidazolidine group (Dmi) group. **(B)** Electrophoretic analyses of SCAN1 (100 nM) reaction products with îligonucleotide substrate containing non-nucleotide insertion (100 nM) (first substrate in panel **A**). Enzyme and substrate were incubated in the reaction buffer (50 mM Tris-HCl, pH 8.0, 50 mM NaCl, 7 mM β-mercaptoethanol) for time indicated at 37°C. The reaction products were separated by 20% polyacrylamide gel electrophoresis under denaturing conditions.

A fluorophore quencher-coupled DNA-biosensor with high sensitivity and specificity for real-time measurement of TDP1 cleavage activity was designed previously in our laboratory (Lebedeva et al., 2015). This biosensor is a short hairpin oligonucleotide with a 1,12-dodecanediol loop, a 5′-fluorescein 5(6)-amide (FAM) fluorophore, and a 3′-BHQ1 (black hole quencher 1) quencher. Specific phosphodiesterase activity of TDP1 is able to remove the quencher from the 3′-end (Lebedeva et al., 2015; Kuznetsov et al., 2017; Komarova et al., 2018; Mamontova et al., 2020). The biosensor contained tetramethyl phosphoryl guanidine (Tmg) group between the 3′-end of DNA and the quencher (Figure 1A, uncleavable Tmg group is designated as p^∗^). This Tmg group is resistant to 3′-phosphodiesterase cleavage. We use it to ensure that the BHQ1-group is not cleaved from the 3′-terminus by the phosphodiesterase activity of TDP1. We used in this work similar hairpin oligonucleotide structures containing different non-nucleotide insertions (X) at the center of the 5′-FAM-GGAAGA-X-TCTTCC-p^∗^-BHQ-3′ chain (Table 1 and Figure 1A; upper oligonucleotide structure). In the previous studies, we found (Lebedeva et al., 2015; Kuznetsov et al., 2017) that TDP1 hydrolyzed phosphodiester bond at the center of the chain 5′ to dodecanediol non-nucleotide insertion. We investigated the TDP1 activity on this substrate by real-time fluorescence intensity measurement and by analysis of the reaction products in polyacrylamide gel with 7 M urea (PAAG). Previously, we have already observed in the gel a weak amount of the second band (Lebedeva et al., 2015; Kuznetsov et al., 2017) in the reaction with hairpin substrate catalyzed by SCAN1. Here, when we analyzed the reaction products of hairpin substrate hydrolysis catalyzed by TDP1 mutant SCAN1 in PAAG, we found two product bands on the gel picture with different mobilities (Figure 1B; P1 and P2). We revealed that the intensity of the second upper product band (P2) decreases in the time course and finally disappears after 30 min (Figure 1B).

**TABLE 1:** Designations and sequences of oligonucleotide substrates.

**No.**	**Designation**	**Sequence, 5**′**–3**′
1	FAM-D-p*BHQ	FAM-GGAAGA**D**TCTTCCp*-BHQ1
2	FAM-DD-p*BHQ	FAM-GGAAGA**DD**TCTTCCp*-BHQ1
3	FAM-Dp*D-p*BHQ	FAM-GGAAGA**D**p*DTCTTCCp*-BHQ1
4	FAM-Dod-p*BHQ	FAM-GGAAGA**Dod**TCTTCCp*-BHQ1
5	FAM-BHQ	FAM-AACGTCAGGGTCTTCC-BHQ1
6	dsFAM-Dod-p*BHQ	5′-FAM-GGAAG**Dod**CCCTGACGTTp*-BHQ1-3′
		3′-CCTTC-**T-**GGGACTGCAA-5′

We assumed that the band P2 could correspond to the oligonucleotide that appears as a result of TDP1 cleavage 3′ to the non-nucleotide insertion (Figure 1A, bold arrow). We supposed that this band eventually disappears, since TDP1 and SCAN1 first hydrolyze the phosphodiesterase bond to the 3′ side and then from the 5′ side of the insert. To test this hypothesis, we synthesized a set of oligonucleotides with different non-nucleotide insertions, including two decandiol residues (Figure 1A). In Figure 1A, the bold arrows indicate the prospective positions of oligonucleotide hydrolysis with TDP1 and SCAN1, giving presumably the second upper product in the picture. The thin fine arrows indicate the hydrolysis position corresponding to the usual lower reaction product (P1). For the entire set of oligonucleotides, the lower products should have identical mobility in the gel, and the mobility of the upper products, presumably, should be different. However, when the products of the reaction were separated in PAAG, we found that the mobility of the second upper product is also the same for oligonucleotides of different structures (Supplementary Figure S1, P2). That is why in the next step, we tried to identify the product of the upper band (P2).

### Identification of the Upper Band Reaction Product by Matrix-Assisted Laser Desorption/Ionization–Mass Spectrometry

We obtained a product corresponding to the upper product band (P2) in an amount required to analyze the composition of the product by mass spectrometry (MS). The oligonucleotide product was purified by gel electrophoresis and chromatography and analyzed by matrix-assisted laser desorption/ionization (MALDI)-MS. The resulting spectrum is shown in Supplementary Figure S2. The spectrum was analyzed, and the main peak of the spectrum corresponded to the lower oligonucleotide product with an “additive” with a molecular weight of 92 g/mol. Such a molecular weight did not correspond to the products that we expected (Figure 1A) but corresponded to the glycerol present in the reaction mixture. Thus, we assumed that the second upper product (P2) of the reaction is the lower product (Pl) with the attached glycerol residue. Since we knew that other phospholipases are capable of catalyzing the transfer reaction, we came up with the conclusion that TDP1 can also catalyze this type of reaction using glycerol as a nucleophile.

**FIGURE 2:**
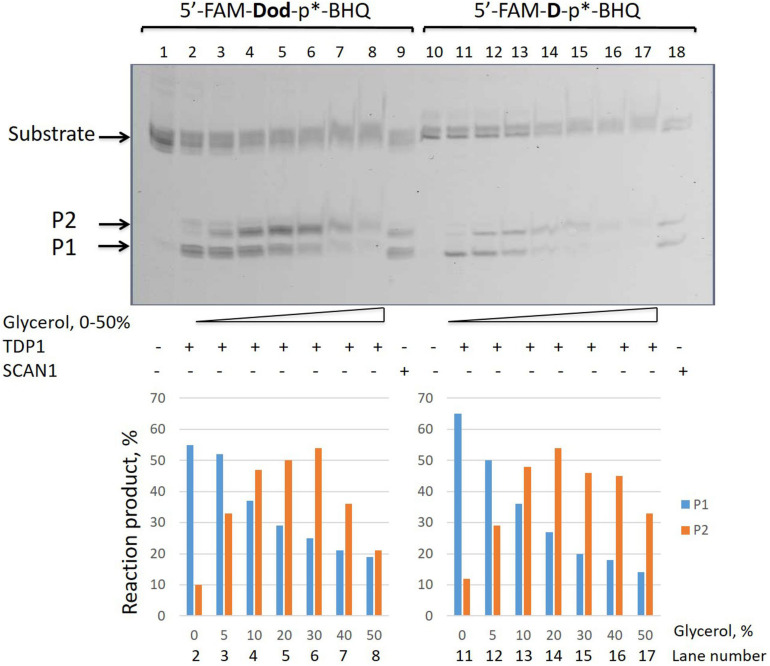
Formation of the upper band tyrosyl-DNA phosphodiesterase 1 (TDP1) reaction product (P2) in the presence of glycerol. Electrophoretic analyses of TDP1 (50 nM) and spinocerebellar ataxia with axonal neuropathy type 1 (SCAN1) (100 nM) reaction products with îligonucleotide substrates containing two different non-nucleotide insertions: D, 1,10-decanediol phosphate; Dod, 1,12-dodecanediol phosphate (100 nM). Enzyme and substrate were incubated in the reaction buffer (50 mM Tris-HCl, pH 8.0, 50 mM NaCl, 7 mM β-mercaptoethanol) for 15 min at 37°C. Schematic representations of DNA substrates (Figure 1 and Table 1) are shown at the top of the gel. The reaction products were separated by 20% polyacrylamide gel electrophoresis under denaturing conditions. The typical representative gel is given on the upper panel. The quantitaion of the reaction products for this gel is on the lower panel. p^∗^ – position of a 1,3-dimethyl-2-(phosphorylimino)imidazolidine group (Dmi) group.

In order to check whether the upper product of the reaction is the lower product with the attached glycerol residue, we added glycerol to the reaction mixture in a concentration of 0–50%. Figure 2 shows that for two substrates with different non-nucleotide insertions, the portion of the upper product increases as compared to the lower product with increasing glycerol concentration.

### Tyrosyl-DNA Phosphodiesterase 1 Excises Glycerol Residue From DNA 3′-End

**FIGURE 3:**
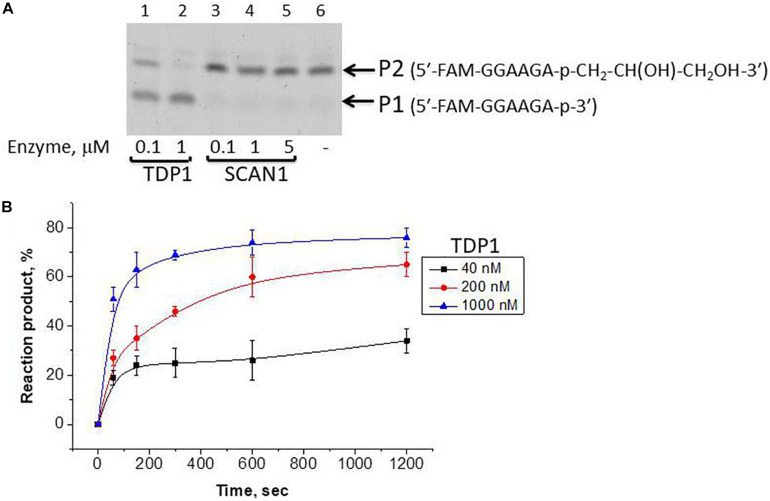
Tyrosyl-DNA phosphodiesterase 1 (TDP1) excises glycerol residue from DNA 3′-end. Enzyme and substrate were incubated in the reaction buffer (50 mM Tris-HCl, pH 8.0, 50 mM NaCl, 7 mM β-mercaptoethanol) for time indicated at 37°C. **(A)** Electrophoretic analyses of TDP1 and spinocerebellar ataxia with axonal neuropathy type 1 (SCAN1) reaction products for 20 min reaction with purified P2 oligonucleotide (100 nM). **(B)** Time dependence of P2 to P1 conversion catalyzed by TDP1 with purified P2 oligonucleotide. Values of the reaction product (%) are the mean (±SD) of three independent experiments. The reaction products were separated by 20% polyacrylamide gel electrophoresis under denaturing conditions.

We took the TDP1 reaction product P2 as a purified oligonucleotide to test the activity of TDP1 and SCAN1 on such an oligonucleotide with a “glycerol” attached at the 3′-end. Figure 3A demonstrates that “glycerol” was efficiently cleaved from the 3′-end by TDP1 (lanes 2, 3), but not SCAN1 in these reaction conditions (lanes 4–8). The efficiency of the product formation (P1) depends on TDP1 concentration (Figure 3A, lanes 2 and 3, and Figure 3B). This TDP1 activity efficiently transforms the substrate up to 50% at a concentration of TDP1 1 μM in 10 s (Figure 3B).

**FIGURE 4:**
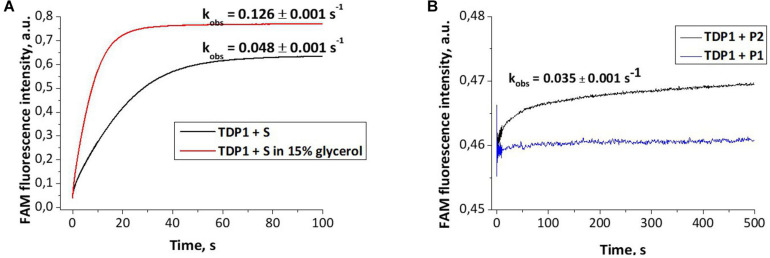
Experimental fluorescence traces revealing the conformational changes during interaction of different oligonucleotides with tyrosyl-DNA phosphodiesterase 1 (TDP1) [(TDP1) = (oligonucleotides) = 1 μM]. **(A)** Interaction of S (FAM- GGAAGA-D-TCTTCC-p*-BHQ) with TDP1. **(B)** Interaction of P1 (5′-FAM-GGAAGA-p-3′) or P2 [5′-FAM-GGAAGA-p-CH2-CH(OH)-CH2OH-3′] purified oligonucleotides with TDP1.

Dye-labeled substrates were subjected to fluorescence resonance energy transfer (FRET) analysis of DNA cleavage reaction in the reaction buffer with or without glycerol. As illustrated in Figure 4A, the changes in FAM fluorescence during the interaction of TDP1 with the FAM-X-p^∗^-BHQ substrate led to an increase in the FRET signal. The increase in FAM fluorescence intensity most likely reflects a release of the cleaved DNA product from the complex with the enzyme that leads to an increase of the distance between FAM and BHQ1 residues. It should be noted that in the presence of 15% glycerol, the observed rate was higher by 2.6-fold then in the case of glycerol-free reaction (0.128 and 0.048 s^–1^, respectively, Figure 4A).

The fluorescence traces obtained for TDP1 upon the interaction with P1 and P2 purified oligonucleotides uncovered an increase in the FAM fluorescence intensity only in the case of P2 (Figure 4B). This difference can indicate that interaction of TDP1 with P1 and P2 oligonucleotides leads to formation of different complexes. Indeed, TDP1 interacts with P2 to produce a catalytic complex that leads to removing of glycerol residue at the 3′-end, whereas P1 represents the final product of an enzyme action. It is interesting to note that the observed rate constants of cleavage of FAM-X-p^∗^-BHQ and P2 are very close (0.048 and 0.035 s^–1^, respectively), indicating that the structure of DNA and nature of the modification are not significantly affected in catalytic reactions.

### Tyrosyl-DNA Phosphodiesterase 1 Catalyzes Transferring Reaction With Other Alcohols and on Other DNA Substrates

We added to the reaction mixture, instead of glycerol, other low-molecular weight organic compounds with hydroxyl groups: alcohols (ethanol, methanol, isopropanol, ethylene glycol, and xylitol) and sugars (glucose and trehalose). The upper second product (P2) was detected in TDP1 and SCAN1 catalyzed reactions with ethanol and methanol (Supplementary Figure S2A), and with a dihydric alcohol–ethylene glycol (Supplementary Figure S2A), and was not detected in reaction with isopropanol and more bulky compounds, polyhydric alcohol xylitol and sugars (data not shown). The formation of the upper product was also demonstrated for the double-stranded oligonucleotide substrate containing the same non-nucleotide insertion (Supplementary Figure S2B) and for single-stranded DNA without the insertion where BHQ was hydrolyzed from 3′-end by TDP1 (Supplementary Figure S2C). We usually use this substrate for TDP1 inhibitor screening (Zakharenko et al., 2019).

**FIGURE 5:**
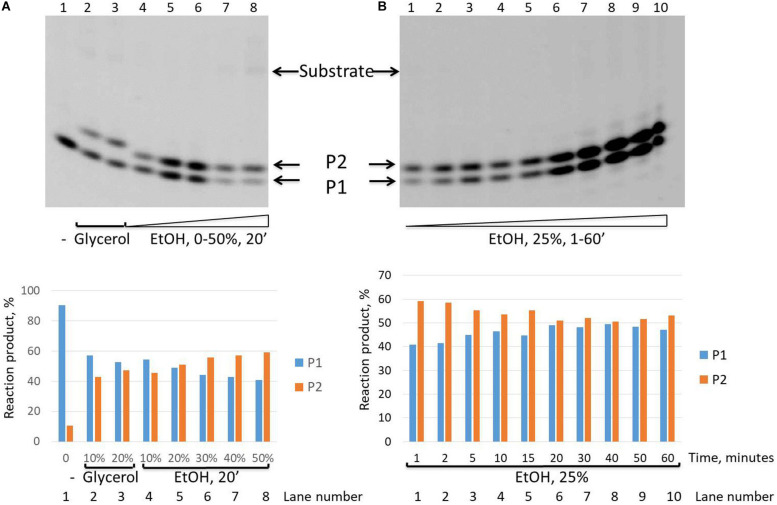
Formation of the upper band tyrosyl-DNA phosphodiesterase 1 (TDP1) reaction product (P2) in the presence of ethanol. Electrophoretic analyses of TDP1 (100 nM) reaction products with îligonucleotide substrate containing non-nucleotide insertion 5′-FAM-D-p*-BHQ (100 nM). Enzyme and substrate were incubated in the reaction buffer (50 mM Tris-HCl, pH 8.0, 50 mM NaCl, 7 mM β-mercaptoethanol) for time indicated at 37°C. **(A)** P2 product formation in dependence of glycerol (lanes 2 and 3) or ethanol (lanes 4–8) concentration. **(B)** Time dependence of P2 product formation. The reaction products were separated by 20% polyacrylamide gel electrophoresis under denaturing conditions. The typical representative gels are given on the upper panels. The quantitaions of the reaction products for the corresponding gels are on the lower panels **(A,B)**.

It is interesting to note that while we revealed that the intensity of the second upper product band (P2) decreases in the time course and finally disappears after 30 min in the presence of the glycerol in the reaction mixture (Figure 2), in the presence of ethanol, this P2 product remained stable up to 60 min of the reaction time course (Figure 5).

### Tyrosyl-DNA Phosphodiesterase 1 Catalyzes Transferring Reaction in the Cell Extracts

We checked whether the substrate (a short hairpin oligonucleotide with non-nucleotide insertion) is cleaved in whole-cell extracts of various types of cells: TK6 (human lymphoblastoid), HEK293 (human embryonic kidney), HeLa (cervical cancer)–WT and TDP1-deficient (TDP1^–/–^), HCT116 (human colon carcinoma), and MCF-7 (human breast adenocarcinoma). The storage buffer of the extracts contained 50% glycerol. The second upper product was present when the substrate was cleaved in cell extracts (Supplementary Figure S4A, lanes 3 and 5–7, and Supplementary Figure S4B, lanes 4, 6, 8, 10, and 11), as in the reaction with the purified recombinant protein SCAN1 (Supplementary Figure S4A, lane 2) and TDP1 in the presence of glycerol (Supplementary Figure S4B, lane 3). At the same time, in WT HeLa, HCT116, and MCF-7, we observed other upper products perhaps corresponding to the other nucleophiles except glycerol presenting in these cell extracts (Supplementary Figure S4A, lanes 6 and 7, Supplementary Figure S4B, lanes 8, 10, and 11). There were no reaction products for TDP1-deficient cell extracts (Supplementary Figure S4A, lane 4, Supplementary Figure S4B, lanes 5, 7, and 9), since hairpin oligonucleotide is a specific TDP1 substrate.

## Discussion

Both phospholipases and nucleases of PLD superfamily hydrolyze phosphodiester bonds via a similar reaction mechanism. They contain paired catalytic histidine and lysine residues within two conserved HKD motifs (HxKxxxxD, where x is any amino acid) (Koonin, 1996; Ponting and Kerr, 1996; Interthal et al., 2001; Selvy et al., 2011; Bruntz et al., 2014). First, crystal structures for PLD enzymes were obtained for a bacterial PLD, *Streptomyces* sp. *strain PMF* (Leiros et al., 2000). Then, protein crystals of other PLD superfamily members have been reported, including endonucleases and several bacterial enzymes, and for human PLD (Bowling et al., 2020; Metrick et al., 2020). PLD enzymes act in two steps. In the first one, the histidine residue from one HKD motif serves as a nucleophile to attack the phosphate group of the substrate. The histidine from the second HKD domain donates proton to the leaving group. Functioning as nucleophiles, the constituent imidazole moieties of the histidines form transient covalent bonds with the substrate, producing an intermediate that can be hydrolyzed next. The second histidine extracts proton from water (or other nucleophile), and the activated molecule hydrolyzes the intermediate (Stuckey and Dixon, 1999; Davies et al., 2002a; Leiros et al., 2000; Selvy et al., 2011; Bruntz et al., 2014).

TDP1 possesses a unique HKD motif that differs from other PLD superfamily members, and its orthologs represent a distinct class within the PLD superfamily (Interthal et al., 2001). TDP1 hydrolyzes DNA adducts containing phosphotyrosyl bond between DNA 3′ phosphate termini and TOP1 residual peptide via two coordinated S_N_2 nucleophilic attacks mediated by the action of two histidine residues like other PLD superfamily enzymes. Initially, the imidazole N1 atom of the His263 residue attacks the phosphotyrosyl bond and then the His493 can act as a general acid, which donates a proton to the tyrosine-containing peptide-leaving group. This results in the formation of a transient phosphoryl imidazole bond between N1 atom of His263 and the 3′-end of the DNA. Hydrolysis of this covalent intermediate is proposed to be carried out by a water molecule that is activated by the His493 residue acting as a general base (Davies et al., 2002a). The only known genetic defect in *Tdp1* gene is the A–G mutation. This homozygous recessive mutation A1478G that results in the substitution His493Arg is associated with the disease SCAN1 (Takashima et al., 2002). It was identified in a genome of the members of one family affected with SCAN1 (Takashima et al., 2002). Cells with a SCAN1 mutation showed increased sensitivity to camptothecin, an anticancer drug, that inhibits TOP1 (Takashima et al., 2002; Hirano et al., 2007). *In vitro* biochemical studies with TDP1 mutant SCAN1 revealed that this mutation reduces enzyme activity ∼25-fold and causes the accumulation of the TDP1–DNA covalent reaction intermediate, supporting the existence of the second S_N_2 reaction step in the TDP1 catalytic mechanism (Interthal et al., 2005a,b).

TDP1 is a DNA repair enzyme that is able to remove a variety of natural and synthetic adducts including stalled TOP1-DNA complexes from 3′ DNA ends preparing the 3′-ends for further processing by DNA polymerases and ligases to complete the repair process and restore the DNA chain (Zakharenko et al., 2019). Other TDP1 substrates can be divided into two groups: small adducts consisting of damaged nucleotides, DNA inserted ribonucleotides, and noncanonical nucleotide/nucleoside analogs and large covalent protein-DNA adducts (Brettrager and van Waardenburg, 2019). Thus, TDP1 is able to remove a variety of adducts from 3′ DNA ends during DNA repair (Dexheimer et al., 2008; Murai et al., 2012; Huang et al., 2013; Dyrkheeva et al., 2018; Brettrager and van Waardenburg, 2019). TDP1 also could be involved in the repair of AP sites in single-stranded genomic DNA regions (Rechkunova et al., 2015).

**FIGURE 6:**
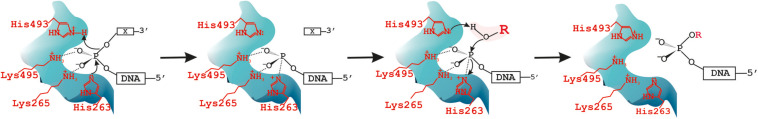
Tyrosyl-DNA phosphodiesterase 1 (TDP1) catalytic cycle: it catalyzes not only hydrolysis but also transferring reaction. X = DNA 3′-end modification [non-nucleotide insertion (Figures 1, 2, 5) or black hole quencher (BHQ) (Supplementary Figure S2B) or glycerol/alcohol (Figure 2)]. R = H in the case of hydrolysis reaction or alcohol: C_2_H_5_-, -C_2_H_4_OH, OHCH_2_-CH(OH)-CH_2_- in the case of transferring reaction catalyzed by TDP1–“transphosphooligonucleotidation.”

Over the past years, we investigated the functions of TDP1 and SCAN1 in the DNA repair process (Zakharenko et al., 2019). It is known that for phospholipases, transphosphatidylation is more favored in the presence of a primary alcohol since generally short-chain primary alcohols are more nucleophilic than water (Bruntz et al., 2014). As proof of the two-step reaction mechanism with the formation of phosphatidyl-enzyme intermediate, the phosphorus chirally labeled substrate with oxygen isotopes at its phosphorus atom was used (Bruzik and Tsai, 1984). Analysis of the stereoconfiguration of the phosphorus before and after the enzymatic reaction by 31P-NMR revealed retention of the configuration, which supported the two-step SN2 reaction mechanism both for transphosphatidylation and hydrolysis reactions catalyzed by cabbage PLD. The formation of intermediate and thus the proof of the two-step reaction mechanism was also given by detecting the phosphatidyl-imidazole intermediate using MS (Orth et al., 2010). We observed two reaction products on the gel in the cleavage of oligonucleotide substrates with non-nucleotide insertion reactions catalyzed by SCAN1 and TDP1 (Kuznetsov et al., 2017). Then we suggested that TDP1 can possess also transferring activity the same as other PLD enzymes. In the present work, we found that the second product with a lower mobility in polyacrylamide gel is generated by WT TDP1 in the presence of high glycerol concentration. We concluded that this product can be generated because glycerol presents in the enzyme solution that was added to the reaction mixture. Next, we purified this oligonucleotide product and confirmed by MALDI-MS that this upper product is a glycerol residue covalently attached to the 3′-end of the lower oligonucleotide product. Thus, we concluded that the second reaction step of TDP1 can be carried out not only by a water molecule but also by the other small nucleophilic molecules, e.g., glycerol, and TDP1 as other PLD family members is able to undergo not only hydrolysis but also transferring reaction. It is known that PLD is able to catalyze synthesis of phosphatidylglycerol by head group exchange of a phosphatidylcholine. The researchers even tried to find the conditions for the preparation of “commercial industrial grades” of phosphatidylglycerol as surfactant/lubricant with unique properties for therapeutic practices for relief of diseases, such as neonatal respiratory distress syndrome (Piazza and Marmer, 2007). We found first that TDP1 as other PLD enzymes catalyzes transferring reaction. According to these results, we proposed a new step of “transphosphooligonucleotidation” analogous to PLDs’ “transphosphatidylation” to the scheme of the reaction catalyzed by TDP1 (Figure 6) and the next step of hydrolysis. The model describing TDP1 catalytic mechanism was suggested in the work of Davies et al. (2002a). In this work, two crystal structures of TDP1 bound to the phosphate transition state analogs, vanadate and tungstate, were solved. It is interesting to note that glycerol molecule from the cryoprotectant solution was found in the structure bound to either vanadate or tungstate inhibitor molecules (Davies et al., 2002a).

We could see the second upper product on the gel not only with glycerol but also with other alcohols including ethanol (Figure 5). It could cause the formation of covalent DNA adducts with different primary alcohol residues in the cell. Such adducts can be accumulated in the conditions of high concentration of alcohol. We demonstrated that glycerol residue was efficiently cleaved from the 3′-end by TDP1 but not by SCAN1 (Figure 3). Thus, in some cases, TDP1 can be regarded not only as a repair enzyme but also as a source of DNA damages especially in the cases of its reduced enzyme activity, for example, in the case of mutations in the *Tdp1* gene. Such damages can make a negative contribution to the stability of cell vitality.

Ethanol is the most frequently used compound with psychoactive and narcotic effects among humans that inhibits the human central nervous system. It is important that it forms solutions in a wide range of proportions with both water and fats probably that is why ethanol has multiple effects on the body. Long-term ethanol consumption can contribute to the development of many diseases, including cardiovascular and cancer. Ethanol can cause oxidative damage to neurons in the brain and their death (Muneer et al., 2011). At the cell nucleus level, ethanol is able to alter access to the cell nucleus by changing the nuclear envelope structure, a double-layered lipid bilayer, penetrated by nuclear pore complexes, thus, the nuclear envelope becomes less permeable for diffusible ions and macromolecules. This could explain altered signaling to and communication with the cell nucleus in the pathophysiology of alcohol abuse (Schäfer et al., 2007). A large body of evidence has shown that alcohol and especially chronic alcohol use can have epigenetic effects, namely, site-selective acetylation, methylation, and phosphorylation in histone, nucleosomal remodeling via histone modifications and DNA methylation (Shukla et al., 2008; Tulisiak et al., 2017; Ciafrè et al., 2019). To date, studies of DNA modifications have primarily looked at global methylation profiles in human liver, brain, and blood, gene-specific methylation profiles in animal models, and methylation changes associated with prenatal ethanol exposure. Our finding of the possibility of direct covalent binding of ethanol to 3′-end of DNA by TDP1 and its mutant form SCAN1 presents a novel insight toward defining the molecular actions of ethanol. Future studies will show the meaning of this fact on cellular and nucleosomal levels. It is known that TDP1 is required for neural homeostasis and acts as a critical survival factor for neuronal development and homeostasis (Katyal et al., 2007; Van Waardenburg, 2016). Though TDP1, like most DNA repair-associated proteins, is not essential for cell viability, TDP1 dysfunction results in SCAN1, a neurodegenerative syndrome. Most researchers investigate TDP1 neuroprotective property with connection to SCAN1 and stabilization of the TDP1 catalytic enzyme-DNA covalent complex, but we could say that in the context of alcoholism and chronic exposure of the nervous system to ethanol, TDP1 neuroprotective property is also important and requires a special detailed study.

## Materials and Methods

### Expression and Purification of Wild-Type and Mutant (SCAN1) Human Tyrosyl-DNA Phosphodiesterase 1 Proteins

The recombinant N-terminally His-tagged TDP1 and the mutant form of TDP1 (SCAN1) with substitution (H493R) were expressed in *Escherichia coli* BL21 (DE3) cells. The plasmids pET16B-TDP1 and pET16B-SCAN1 were kindly provided by Dr. K. W. Caldecott, University of Sussex, United Kingdom, and by Dr. S. El-Khamisy, University of Sheffield, United Kingdom. Plasmids were transformed into BL21 cells by electroporation, and the cells were grown in LB medium at pH 7.5 with 100 mg/ml ampicillin at 30°C. Two hours after induction with 1 mM isopropyl β-d-1-thiogalactopyranoside (IPTG), cells were harvested. Cell pellets were thawed on ice, resuspended in binding buffer (0.5 M NaCl, 5% glycerol, 20 mM Tris-HCl, pH 8.0, mixture of protease inhibitors), and broken by sonication. After centrifugation, 10 mM imidazole was added to the supernatant. The Ni Sepharose column (GE Healthcare, United Kingdom) was washed with binding buffer (0.5 M NaCl, 10 mM imidazole, 20 mM Tris-HCl, pH 8.0). Elution of the proteins was carried out with elution buffer (0.5 M NaCl, 500 mM imidazole, 20 mM Tris-HCl, pH 8.0, protease inhibitors), and the eluate was loaded to the heparin Sepharose column (GE Healthcare, United Kingdom). Elution of the proteins was carried out with NaCl gradient 0.1–1 M in 20 mM Tris-HCl pH 8.0 with protease inhibitors. The proteins TDP1 and SCAN1 were stored in 50 mM NaCl, 50 mM Tris-HCl pH 8.0, 1 mM ethylenediaminetetraacetic acid (EDTA), 2 mM dithiothreitol (DTT), and 50% glycerol at -20°C. The enzyme samples were estimated to be more than 90% pure. Enzyme concentrations were estimated by Bradford assay. Coomassie-stained protein gels are shown in Supplementary Figure S5.

### Oligonucleotide Substrate Preparation

Oligonucleotides were synthesized on a Biosset ASM-800 automated DNA synthesizer (Russia) on 200-nmol scale using β-cyanoethyl phosphoramidite chemistry. Here, 1,10-decanediol residue (D) and 1,12-dodecanediol residue (Dod) were introduced via the corresponding dimethoxytrityl phosphoramidite prepared as described previously (Durand et al., 1990). 5(6)-FAM phosphoramidite for FAM labeling and a solid support 3′-BHQ-1 CPG for attachment of a Black Hole QuencherTM BHQ-1 residue were generous gifts of Dr. Vladimir Ryabinin (ICBFM, Novosibirsk). Incorporation of a 1,3-dimethyl-2-(phosphorylimino)imidazolidine (Dmi) group was performed as described (Stetsenko et al., 2014). After the completion of solid-phase synthesis, polymer support from the column was transferred to a plastic tube and treated with 200 μl of concentrated (ca. 25%) aqueous ammonia solution per 5 mg of support at 55°C for 16 h. After deprotection, the supernatant was evaporated in vacuo using a Thermo Fisher Scientific SpeedVac concentrator (United States), 400 μl of 20 mM triethylammonium acetate (pH 7.0) was added, and supernatant was removed by centrifugation. Oligonucleotides were purified by reverse-phased (RP) high-performance liquid chromatography (HPLC) on an Agilent 1200 series HPLC system (United States) equipped with a Zorbax SB-C18 (5 μm) column (4.6 mm × 150 mm) using a gradient of acetonitrile from 0 to 40% in 0.02 M triethylammonium acetate pH 7.0 for 30 min, flow rate 2 ml/min. Denaturing gel electrophoresis in 20% polyacrylamide gel was used to check the purity of oligonucleotides with band visualization by staining with Stains-All (Sigma). Molecular masses of modified oligonucleotides were confirmed by electrospray ionization (ESI) mass spectra recorded on an Agilent G6410A LC-MS/MS triple quadrupole ESI mass spectrometer (United States) in the MS scan mode with negative ion detection. The oligonucleotides were dissolved to 0.1 mM concentration in 20 mM triethylammonium acetate containing 60% acetonitrile for direct injection (10 μl). Elution was made by 80% acetonitrile in isocratic mode, flow rate 0.1 ml/min. Default parameters for ESI and MS were used for all the experiments: nebulizer gas pressure was 30 psi (207 kPa), drying gas (nitrogen) flow rate was 9 L/min and temperature 340°C, capillary voltage was 4,000 V, detected mass range was from m/z 105 to 1,600. Molecular masses of oligonucleotides were calculated uzing experimental m/z values, obtained for each sample.

Molecular masses of modified oligonucleotides were confirmed byMALDI-time of flight (MALDI-TOF) mass spectra recorded in either negative or positive ion mode on a Bruker Reflex III Autoflex Speed mass spectrometer (Germany) using 3-hydroxypicolinic acid as a matrix.

Structures of the chemical modifications used in this study are depicted in Supplementary Figure S6. Sequences and ESI MS data of the oligonucleotides are given in Table 1.

BHQ1, Black Hole Quencher 1; FAM, 5(6)-carboxyfluorescein label; non-nucleotide insertions (X); D, 1,10-decanediol phosphate; Dod, 1,12-dodecanediol phosphate; p^∗^, position of a 1,3-dimethyl-2-(phosphorylimino)imidazolidine group (Dmi) group. See Supplementary Figure S6 for all the structures.

### Gel-Based Tyrosyl-DNA Phosphodiesterase 1 Assay

TDP1 gel-based assays were performed using 100 nM substrate incubated with the indicated amount of recombinant human TDP1 or SCAN1 or cell extract for time indicated at 37°C in a buffer containing 50 mM Tris-HCl, pH 8.0, 50 mM NaCl, and 7 mM β-mercaptoethanol. To avoid the formation of the upper oligonucleotide product with attached glycerol residue from the enzyme solution, we purified TDP1 and SCAN1 solution on the SuperSpin Desaltor (Biotoolmics) columns. Reactions were terminated by the addition of gel loading buffer (TBE, 10% formamide, 7 M carbamide, 0.1% xylene cyanol, and 0.1% bromophenol blue, 20 mM EDTA). The samples were heated before loading at 90°C for 7 min. The products were analyzed by electrophoresis in a 20% denaturing polyacrylamide gel with 7 M urea. Gel images were scanned using a Typhoon FLA 9500 (GE Healthcare, United Kingdom) and calculated using a QuantityOne 4.6.7 software. The reaction product yields (%) were calculated as the percentage of the fluorescent signal of the product band to the total signal of the bands in the lane.

### Stopped-Flow Fluorescence Measurements

Kinetic studies of the reactions were carried out using an SX.18MV stopped-flow spectrometer (Applied Photophysics, United Kingdom). The efficiency of energy transfer in the FAM/BHQ1 FRET pair was recorded with fluorescence excitation in the FAM dye at 494 nm. Fluorescence of the FAM dye was recorded at wavelengths more than 515 nm using an OG-515 filter (Schott, Germany). The dead time of the device is 1.4 ms. Each kinetic curve was averaged over at least four experimental curves. All experiments were performed at 37°C in the buffer solution containing 50 mM Tris-HCl, pH 8.0, 50 mM NaCl. In the course of FRET experiments, the enzyme and DNA concentrations were 1.0 μM.

The solution of TDP1 was placed in one instrument’s syringe and rapidly mixed in the reaction chamber with the substrate from another syringe. The reported concentrations of reactants are those in the reaction chamber after mixing. Typically, each trace shown in the figures is the average of four or more fluorescence traces recorded in individual experiments. FRET analysis revealed changes in the distance between the dye and quencher in the processes of DNA cleavage reaction.

## Data Availability Statement

The original contributions presented in the study are included in the article/Supplementary Material, further inquiries can be directed to the corresponding author/s.

## Author Contributions

ND and NL did all the experiments except stopped flow. ND and RA purified the recombinant proteins. MK synthesized the oligonucleotides. RA and MK did MALDI-MS decoding. AK and NK did the stopped flow experiments. ND, RA, NR, and OL designed the study. All authors contributed to the results discussion and manuscript writing.

## Conflict of Interest

The authors declare that the research was conducted in the absence of any commercial or financial relationships that could be construed as a potential conflict of interest.

## References

[B2] Bowling F. Z. Salazar C. M. Bell J. A. Huq T. S. Frohman M. A. Airola M. V. (2020). Crystal structure of human PLD1 provides insight into activation by PI(4,5)P2 and RhoA. *Nat. Chem. Biol.* 16 400–407. 10.1038/s41589-020-0499-8 32198492 PMC7117805

[B3] Brettrager E. J. van Waardenburg R. C. A. M. (2019). Targeting Tyrosyl-DNA phosphodiesterase I to enhance toxicity of phosphodiester linked DNA-adducts. *Cancer Drug Resist.* 2 1153–1163. 10.20517/cdr.2019.91 31875206 PMC6929713

[B4] Bruntz R. C. Lindsley C. W. Brown H. A. (2014). Phospholipase D signaling pathways and phosphatidic acid as therapeutic targets in cancer. *Pharmacol. Rev.* 66 1033–1079. 10.1124/pr.114.009217 25244928 PMC4180337

[B5] Bruzik K. Tsai M. D. (1984). Phospholipids chiral at phosphorus. Synthesis of chiral phoshatidylcholine and stereochemistry of phospholipase D. *Biochemistry* 23 1656–1661. 10.1021/bi00303a012 6722117

[B6] Ciafrè S. Carito V. Ferraguti G. Greco A. Chaldakov G. N. Fiore M. (2019). How alcohol drinking affects our genes: an epigenetic point of view. *Biochem. Cell Biol.* 97 345–356. 10.1139/bcb-2018-0248 30412425

[B7] Davies D. R. Interthal H. Champoux J. J. Hol W. G. J. (2002a). Insights into substrate binding and catalytic mechanism of human tyrosyl-DNA phosphodiesterase (TDP1) from vanadate and tungstate-inhibited structures. *J. Mol. Biol.* 324 917–932. 10.1016/s0022-2836(02)01154-3 12470949

[B8] Davies D. R. Interthal H. Champoux J. J. Hol W. G. J. (2002b). The crystal structure of human tyrosyl-DNA phosphodiesterase. Tdp1. *Structure* 10 237–248. 10.1016/s0969-2126(02)00707-4 11839309

[B9] Dexheimer T. S. Antony S. Marchand C. Pommier Y. (2008). Tyrosyl-DNA phosphodiesterase as a target for anticancer therapy. *Anticancer Agents Med. Chem.* 8 381–389. 10.2174/187152008784220357 18473723 PMC2443942

[B10] Durand M. Chevrie K. Chassignol M. Thuong N. T. Maurizot J. C. (1990). Circular dichroism studies of an oligodeoxyribonucleotide containing a hairpin loop made of a hexaethylene glycol chain: conformation and stability. *Nucleic Acids Res.* 18 6353–6359. 10.1093/nar/18.21.6353 2243780 PMC332506

[B11] Dyrkheeva N. S. Lebedeva N. A. Sherstyuk Y. V. Abramova T. V. Silnikov V. N. Lavrik O. I. (2018). Excision of carbohydrate-modified dNMP analogues from DNA 3′ end by human apurinic/apyrimidinic endonuclease 1 (APE1) and Tyrosyl-DNA Phosphodiesterase 1 (TDP1). *Mol. Biol.* 52 1066–1073. 10.1134/S002689841806006X 30633249

[B12] Frohman M. A. (2015). The phospholipase D superfamily as therapeutic targets. *Trends Pharmacol Sci.* 36 137–144. 10.1016/j.tips.2015.01.001 25661257 PMC4355084

[B13] Hawkins A. J. Subler M. A. Akopiants K. Wiley J. L. Taylor S. M. Rice A. C. (2009). In vitro complementation of TDP1 deficiency indicates a stabilized enzyme-DNA adduct from tyrosyl but not glycolate lesions as a consequence of the SCAN1 mutation. *DNA Repair.* 8 654–663. 10.1016/j.dnarep.2008.12.012 19211312 PMC2844109

[B14] Hirano R. Interthal H. Huang C. Nakamura T. Deguchi K. Choi K. (2007). Spinocerebellar ataxia with axonal neuropathy: consequence of a Tdp1 recessive neomorphic mutation? *EMBO J.* 26 4732–4743. 10.1038/sj.emboj.7601885 17948061 PMC2080798

[B15] Huang S. Y. Murai J. Dalla Rosa I. Dexheimer T. S. Naumova A. Gmeiner W. H. (2013). TDP1 repairs nuclear and mitochondrial DNA damage induced by chain-terminating anticancer and antiviral nucleoside analogs. *Nucleic Acids Res.* 41 7793–7803. 10.1093/nar/gkt483 23775789 PMC3763526

[B16] Inamdar K. V. Pouliot J. J. Zhou T. Lees-Miller S. P. Rasouli-Nia A. Povirk L. F. (2002). Conversion of phosphoglycolate to phosphate termini on 3′ overhangs of DNA double strand breaks by the human tyrosyl-DNA phosphodiesterase hTdp1. *J. Biol. Chem.* 277 27162–27168. 10.1074/jbc.M204688200 12023295

[B17] Interthal H. Chen H. J. Champoux J. J. (2005a). Human TDP1 cleaves a broad spectrum of substrates, including phosphoamide linkages. *J. Biol. Chem.* 280 36518–36528. 10.1074/jbc.M508898200 16141202 PMC1351008

[B18] Interthal H. Chen H. J. Kehl-Fie T. E. Zotzmann J. Leppard J. B. Champoux J. J. (2005b). SCAN1 mutant TDP1 accumulates the enzyme–DNA intermediate and causes camptothecin hypersensitivity. *EMBO J.* 24 2224–2233. 10.1038/sj.emboj.7600694 15920477 PMC1150888

[B19] Interthal H. Pouliot J. J. Champoux J. J. (2001). The tyrosyl-DNA phosphodiesterase TDP1 is a member of the phospholipase D superfamily. *Proc. Natl. Acad. Sci. U.S.A.* 98 12009–12014. 10.1073/pnas.211429198 11572945 PMC59758

[B20] Katyal S. el-Khamisy S. F. Russell H. R. Li Y. Ju L. Caldecott K. W. (2007). TDP1 facilitates chromosomal single-strand break repair in neurons and is neuroprotective in vivo. *EMBO J.* 26 4720–4731. 10.1038/sj.emboj.7601869 17914460 PMC2080805

[B21] Komarova A. O. Drenichev M. S. Dyrkheeva N. S. Kulikova I. V. Oslovsky V. E. Zakharova O. D. (2018). Novel group of tyrosyl-DNA-phosphodiesterase 1 inhibitors based on disaccharide nucleosides as drug prototypes for anti-cancer therapy. *J. Enzyme. Inhib. Med. Chem.* 33 1415–1429. 10.1080/14756366.2018.1509210 30191738 PMC6136360

[B22] Koonin E. V. (1996). A duplicated catalytic motif in a new superfamily of phosphohydrolases and phospholipid synthases that includes poxvirus envelope proteins. *Trends Biochem. Sci.* 21 242–243. 10.1016/s0968-0004(96)30024-8 8755242

[B23] Kuznetsov N. A. Lebedeva N. A. Kuznetsova A. A. Rechkunova N. I. Dyrkheeva N. S. Kupryushkin M. S. (2017). Pre-steady state kinetics of DNA binding and abasic site hydrolysis by tyrosyl-DNA phosphodiesterase 1. *J. Biomol. Struct. Dyn.* 35 2314–2327. 10.1080/07391102.2016.1220331 27687298

[B24] Lebedeva N. A. Anarbaev R. O. Kupryushkin M. S. Rechkunova N. I. Pyshnyi D. V. Stetsenko D. A. (2015). Design of a new fluorescent oligonucleotide-based assay for a highly specific real-time detection of apurinic/apyrimidinic site cleavage by Tyrosyl-DNA Phosphodiesterase 1. *Bioconjug. Chem.* 26 2046–2053. 10.1021/acs.bioconjchem.5b00451 26335988

[B25] Lebedeva N. A. Rechkunova N. I. El-Khamisy S. F. Lavrik O. I. (2012). Tyrosyl-DNA phosphodiesterase 1 initiates repair of apurinic/apyrimidinic sites. *Biochimie* 94 1749–1753. 10.1016/j.biochi.2012.04.004 22522093 PMC3778944

[B26] Lebedeva N. A. Rechkunova N. I. Ishchenko A. A. Saparbaev M. Lavrik O. I. (2013). The mechanism of human tyrosyl-DNA phosphodiesterase 1 in the cleavage of AP site and its synthetic analogs. *DNA Repair* 12 1037–1042. 10.1016/j.dnarep.2013.09.008 24183900

[B27] Lebedeva N. A. Rechkunova N. I. Lavrik O. I. (2011). AP-site cleavage activity of tyrosyl-DNA phosphodiesterase 1. *FEBS Lett.* 585 683–686. 10.1016/j.febslet.2011.01.032 21276450

[B28] Leiros I. Secundo F. Zambonelli C. Servi S. Hough E. (2000). The first crystal structure of a phospholipase D. *Structure* 8 655–667. 10.1016/s0969-2126(00)00150-7 10873862

[B29] Mamontova E. M. Zakharenko A. L. Zakharova O. D. Dyrkheeva N. S. Volcho K. P. Reynisson J. (2020). Identification of novel inhibitors for the tyrosyl-DNA-phosphodiesterase 1 (Tdp1) mutant SCAN1 using virtual screening. *Bioorg. Med. Chem.* 28:115234. 10.1016/j.bmc.2019.115234 31831297

[B30] Metrick C. M. Peterson E. A. Santoro J. C. Enyedy I. J. Murugan P. Chen T. (2020). Human PLD structures enable drug design and characterization of isoenzyme selectivity. *Nat. Chem. Biol.* 16 391–399. 10.1038/s41589-019-0458-4 32042197

[B31] Muneer P. M. Alikunju S. Szlachetka A. M. Haorah J. (2011). Inhibitory effects of alcohol on glucose transport across the blood-brain barrier leads to neurodegeneration: preventive role of acetyl-L-carnitine. *Psychopharmacology* 214 707–718. 10.1007/s00213-010-2076-4 21079922 PMC3055928

[B32] Murai J. Huang S. Y. Das B. B. Dexheimer T. S. Takeda S. Pommier Y. (2012). Tyrosyl-DNA phosphodiesterase 1 (TDP1) repairs DNA damage induced by topoisomerases I and II and base alkylation in vertebrate cells. *J. Biol. Chem.* 287 12848–12857. 10.1074/jbc.M111.333963 22375014 PMC3339927

[B33] Nitiss K. C. Malik M. He X. White S. W. Nitiss J. L. (2006). Tyrosyl-DNA phosphodiesterase (Tdp1) participates in the repair of Top2-mediated DNA damage. *Proc. Natl. Acad. Sci. U.S.A.* 103 8953–8958. 10.1073/pnas.0603455103 16751265 PMC1482547

[B34] Orth E. S. Brandão T. A. S. Souza B. S. Pliego J. R. Vaz B. G. Eberlin M. N. (2010). Intramolecular catalysis of phosphodiester hydrolysis by two imidazoles. *J. Am. Chem. Soc.* 132 8513–8523. 10.1021/ja1034733 20509675

[B35] Peng X. Frohman M. A. (2012). Mammalian phospholipase D physiological and pathological roles. *Acta Physiol.* 204 219–226. 10.1111/j.1748-1716.2011.02298.x 21447092 PMC3137737

[B36] Piazza G. J. Marmer W. N. (2007). Conversion of Phosphatidylcholine to Phosphatidylglycerol with Phospholipase D and Glycerol. *J. Am. Oil Chem. Soc.* 84 645–651. 10.1007/s11746-007-1081-1

[B37] Ponting C. P. Kerr I. D. (1996). A novel family of phospholipase D homologues that includes phospholipid synthases and putative endonucleases: identification of duplicated repeats and potential active site residues. *Protein Sci.* 5 914–922. 10.1002/pro.5560050513 8732763 PMC2143407

[B38] Pouliot J. J. Yao K. C. Robertson C. A. Nash H. A. (1999). Yeast gene for a Tyr-DNA phosphodiesterase that repairs topoisomerase I complexes. *Science* 286 552–555. 10.1126/science.286.5439.552 10521354

[B39] Rass U. Ahel I. West S. C. (2007). Defective DNA repair and neurodegenerative disease. *Cell* 130 991–1004. 10.1093/nar/gkaa489 17889645

[B40] Raymond A. C. Staker B. L. Burgin A. B. Jr. (2005). Substrate specificity of tyrosyl-DNA phosphodiesterase I (Tdp1). *J. Biol. Chem.* 280 22029–22035. 10.1074/jbc.M502148200 15811850

[B41] Rechkunova N. I. Lebedeva N. A. Lavrik O. I. (2015). Tyrosyl-DNA phosphodiesterase 1 is a new player in repair of apurinic/apyrimidinic sites. *Bioorg. Khim.* 41 531–538. 10.1134/s106816201505012x 26762090

[B42] Schäfer C. Ludwig Y. Shahin V. Kramer A. Carl P. Schillers H. (2007). Ethanol alters access to the cell nucleus. *Cell Mol. Physiol.* 453 809–818. 10.1007/s00424-006-0165-3 17043811

[B43] Selvy P. E. Lavieri R. R. Lindsley C. W. Brown H. A. (2011). Phospholipase D: enzymology. functionality, and chemical modulation. *Chem. Rev.* 111 6064–6119. 10.1021/cr200296t 21936578 PMC3233269

[B44] Shukla S. D. Velazquez J. French S. W. Lu S. C. Ticku M. K. Zakhari S. (2008). Emerging role of epigenetics in the actions of alcohol. *Alcohol. Clin. Exp. Res.* 32 1525–1534. 10.1111/j.1530-0277.2008.00729.x 18616668

[B45] Stetsenko D. A. Kupryushkin M. S. Pyshnyi D. V. (2014). Modified oligonucleotides and methods for their synthesis. *International Patent No. WO2016028187A1.*

[B46] Stuckey J. A. Dixon J. E. (1999). Crystal structure of a phospholipase D family member. *Nat. Struct. Biol.* 6 278–284. 10.1038/6716 10074947

[B47] Takashima H. Boerkoel C. F. John J. Saifi G. M. Salih M. A. Armstrong D. (2002). Mutation of TDP1, encoding a topoisomerase I-dependent DNA damage repair enzyme, in spinocerebellar ataxia with axonal neuropathy. *Nat. Genet.* 32 267–272. 10.1038/ng987 12244316

[B48] Tulisiak C. T. Harris R. A. Ponomarev I. (2017). DNA modifications in models of alcohol use disorders. *Alcohology* 60 19–30. 10.1016/j.alcohol.2016.11.004 27865607 PMC5420490

[B49] Van Waardenburg R. C. A. M. (2016). Tyrosyl-DNA Phosphodiesterase I a critical survival factor for neuronal development and homeostasis. *J. Neurol. Neuromed.* 1 25–29. 10.29245/2572.942x/2016/5.1048 PMC5064944 27747316

[B50] Yang S. W. Burgin A. B. Jr. Huizenga B. N. Robertson C. A. Yao K. C. Nash H. A. (1996). A eukaryotic enzyme that can disjoin dead-end covalent complexes between DNA and type I topoisomerases. *Proc. Natl. Acad. Sci. U.S.A.* 93 11534–11539. 10.1073/pnas.93.21.11534 8876170 PMC38092

[B51] Zakharenko A. Dyrkheeva N. Lavrik O. (2019). Dual DNA topoisomerase 1 and tyrosyl-DNA phosphodiesterase 1 inhibition for improved anticancer activity. *Med. Res. Rev.* 39 1427–1441. 10.1002/med.21587 31004352

[B52] Zhang H. Xiong Y. Chen J. (2020). DNA-protein cross-link repair: what do we know now? *Cell Biosci.* 10:3. 10.1186/s13578-019-0366-z 31921408 PMC6945406

[B53] Zhou T. Lee J. W. Tatavarthi H. Lupski J. R. Valerie K. Povirk L. F. (2005). Deficiency in 3′-phosphoglycolate processing in human cells with a hereditary mutation in tyrosyl-DNA phosphodiesterase (TDP1). *Nucleic Acids Res.* 33 289–297. 10.1093/nar/gki170 15647511 PMC546157

