# Supplementary material for: Human Tyrosyl-DNA Phosphodiesterase 1 Possesses Transphosphooligonucleotidation Activity With Primary Alcohols
Source: Front Cell Dev Biol. 2020 Dec 23;8:604732. doi: 10.3389/fcell.2020.604732 (PMC7786179; doi:10.3389/fcell.2020.604732)
Supplement: Supplementary file 1 [file Data_Sheet_1.DOCX]

Supplementary Material

**
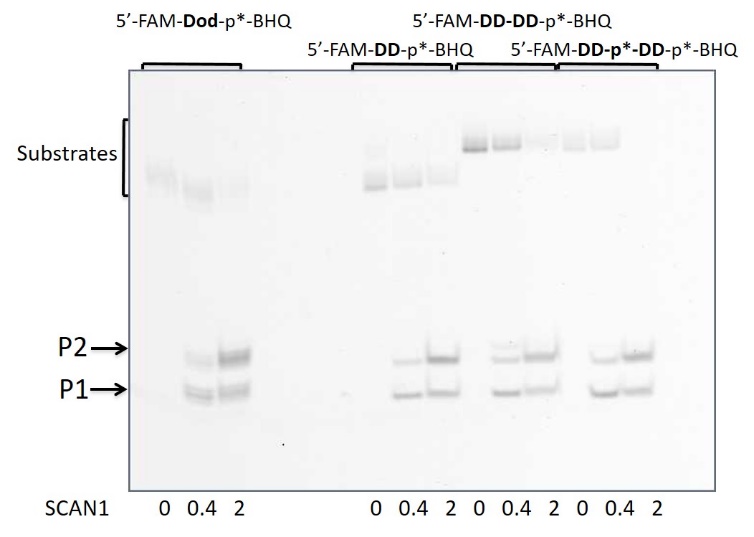
**

**Figure S1**. Electrophoretic analyses of SCAN1 (100 nM) reaction products with оligonucleotide substrates containing different non-nucleotide insertions (Table 1): D – 1,10-decanediol phosphate, Dod – 1,12-dodecanediol phosphate (100 nM). BHQ1 – Black Hole Quencher 1; FAM – 5(6)-carboxyfluorescein label; non-nucleotide insertions (X); p* – 1,3-dimethyl-2-(phosphorylimino)imidazolidine group (Dmi) group. The reaction products were separated by 20% polyacrylamide gel electrophoresis under denaturing conditions.


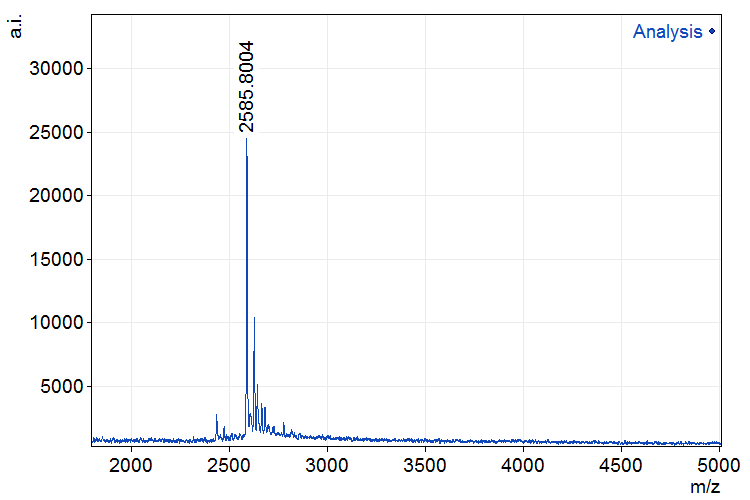


**Figure S2**. MALDI TOF mass spectra of the purified oligonucleotide product (P2) of TDP1 catalyzed reaction with oligonucleotide 5'-FAM-GGAAGA-D-TCTTCC-p*-BHQ-3' containing non-nucleotide insertion (D – 1,10-decanediol phosphate).

***
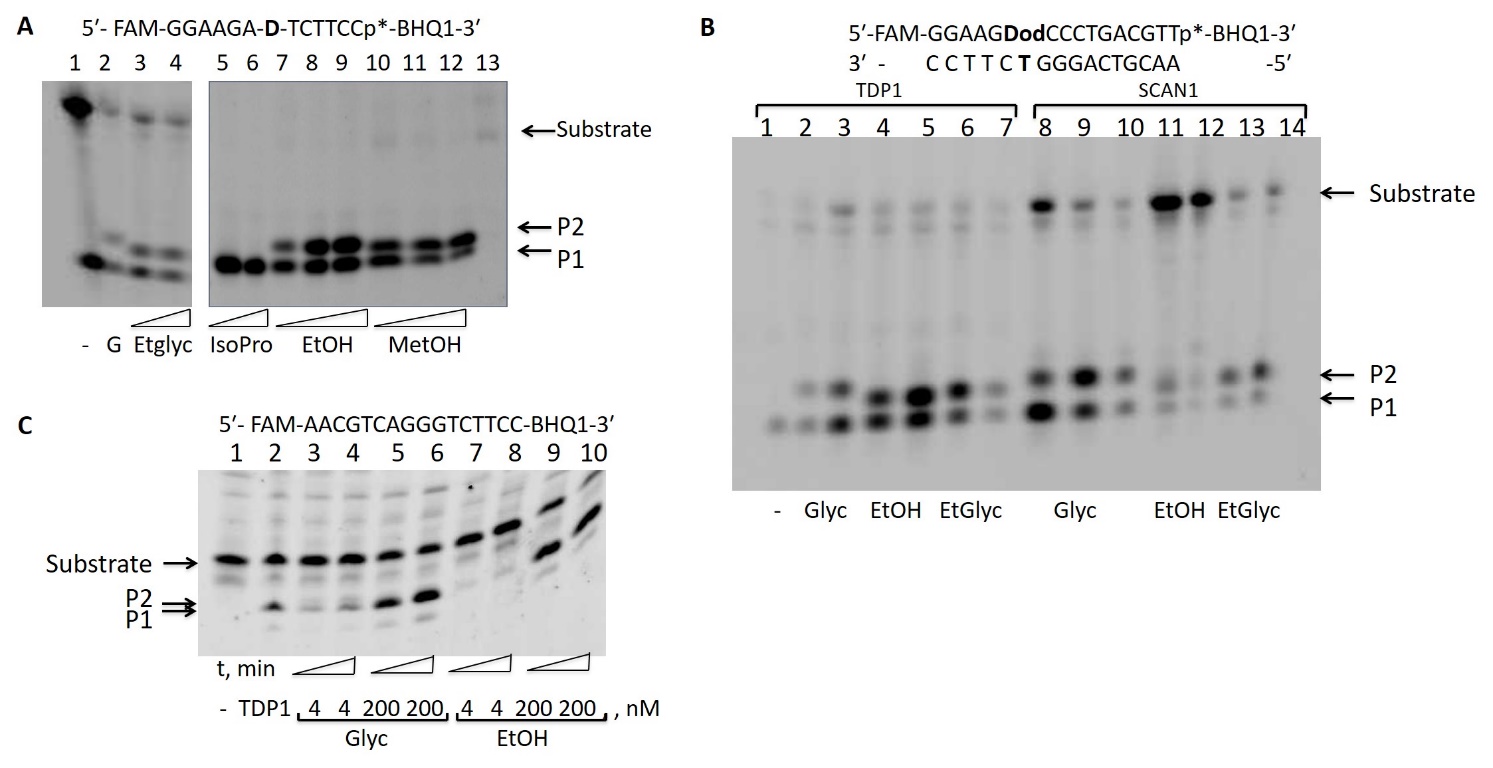
***

**Figure S3**. Electrophoretic analyses of TDP1 (50 nM, A-C) and SCAN1 (100 nM, B) reaction products with different оligonucleotide substrates (100 nM) containing non-nucleotide insertions (A and B) and FAM-AACGTCAGGGTCTTCC-BHQ1 (C) (Table 1). D – 1,10-decanediol phosphate; Dod – 1,12-dodecanediol phosphate; G or Glyc – glycerol; Etglyc – ethylene glycol; IsoPro – isopropanol. The reaction products were separated by 20% polyacrylamide gel electrophoresis under denaturing conditions.


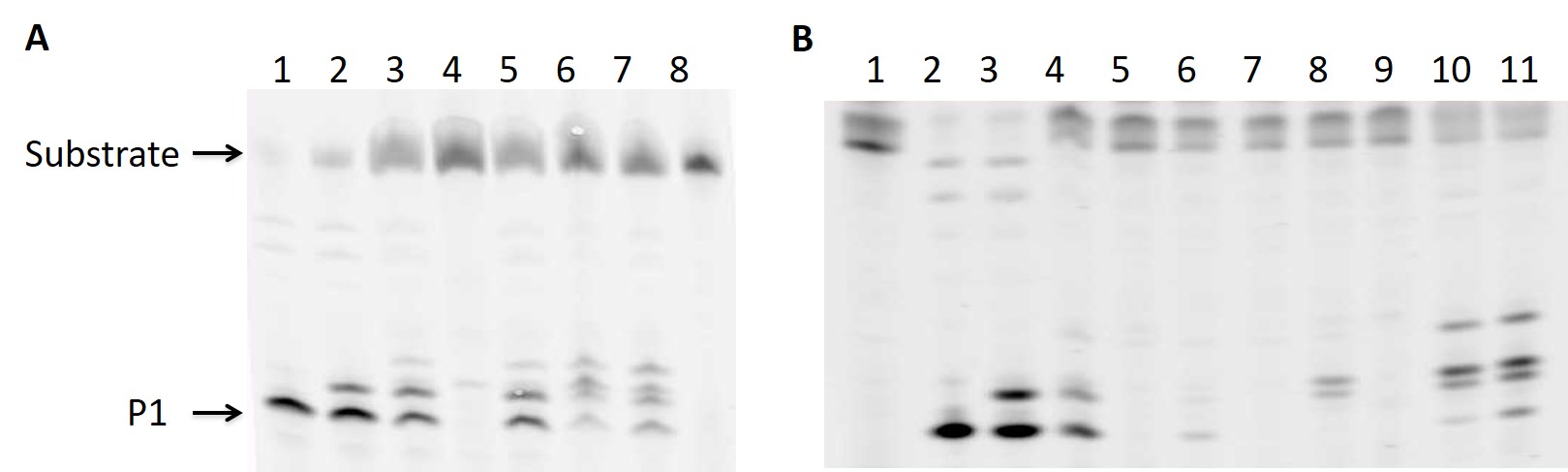


**Figure S4**. Electrophoretic analyses of TDP1 reaction products with purified proteins TDP1/SCAN1 or cell extracts and оligonucleotide substrate containing non-nucleotide insertion 5′-FAM-D-p*-BHQ (100 nM). The reaction products were separated by 20% polyacrylamide gel electrophoresis under denaturing conditions. (A) Lanes: 1. purified TDP1; 2. purified SCAN1; 3. ТК6 (human lymphoblastoid) cell extract; 4. Tdp1 -/- ТК6 cell extract; 5. ТК6 cell extract; 6. НСТ (human colon carcinoma) cell extract; 7. MCF (human breast adenocarcinoma) cell extract; 8. No enzyme. (B) Lanes: 1. No enzyme; 2. purified TDP1; 3. purified TDP1 with 12.5% glycerol; 4. ТК6 cell extract; 5. Tdp1 -/- ТК6 cell extract; 6. HEK293 (human embryonic kidney) cell extract; 7. Tdp1 -/- HEK293 cell extract; 8. HeLa (cervical cancer)) cell extract; 9. Tdp1 -/- HeLa cell extract; 10. НСТ cell extract; 11. MCF cell extract.


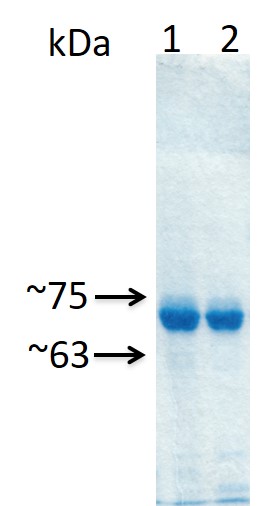


**Figure S5**. SDS-PAGE analysis of the purification of TDP1 (lane 1) and SCAN1 (lane 2).

**Figure S6**. Structures of oligonucleotide chemical modifications used in the study.
